# Genome-Wide Analysis of Disordered Eating Behavior in the Mexican Population

**DOI:** 10.3390/nu14020394

**Published:** 2022-01-17

**Authors:** José Jaime Martínez-Magaña, Sandra Hernandez, Ana Rosa Garcia, Valeria Cardoso-Barajas, Emmanuel Sarmiento, Beatriz Camarena, Alejandro Caballero, Laura Gonzalez, Jorge Ameth Villatoro-Velazquez, Maria Elena Medina-Mora, Marycarmen Bustos-Gamiño, Clara Fleiz-Bautista, Carlos Alfonso Tovilla-Zarate, Isela Esther Juárez-Rojop, Humberto Nicolini, Alma Delia Genis-Mendoza

**Affiliations:** 1 Laboratorio de Genómica de Enfermedades Psiquiátricas y Neurodegenerativas, Instituto Nacional de Medicina Genómica, Mexico City 14610, Mexico; jimy.10.06@gmail.com; 2 Laboratorio de Farmacogenética, Instituto Nacional de Psiquiatría Ramón de la Fuente Muñiz, Mexico City 14370, Mexico; sanher90@hotmail.com (S.H.); camare@imp.edu.mx (B.C.); 3 Unidad de Investigación, Hospital Psiquiátrico Infantil Juan N. Navarro, Mexico City 14080, Mexico; anarosagarciab@gmail.com (A.R.G.); morganakvm@hotmail.com (V.C.-B.); emmanuel.sarmiento@salud.gob.mx (E.S.); 4 Unidad de Trastornos Alimenticios, Instituto Nacional de Psiquiatría Ramón de la Fuente Muñiz, Mexico City 14370, Mexico; alexcarom@hotmail.com (A.C.); macias@imp.edu.mx (L.G.); 5 Unidad de Análisis de Datos y Encuestas, Instituto Nacional de Psiquiatría Ramón de la Fuente Muñiz, Mexico City 14370, Mexico; ameth@imp.edu.mx (J.A.V.-V.); metmmora@gmail.com (M.E.M.-M.); naomi_gam@hotmail.com (M.B.-G.); clarafleiz@yahoo.com.mx (C.F.-B.); 6 División Académica Multidisciplinaria de Comalcalco, Universidad Juárez Autónoma de Tabasco, Comalcalco 86654, Mexico; alfonso_tovillaz@yahoo.com.mx; 7 División de Ciencias de la Salud, Universidad Juárez Autónoma de Tabasco, Villahermosa 86100, Mexico; iselajuarezrojop@hotmail.com

**Keywords:** feeding and eating disorder, genome-wide association study, methylation quantitative trait loci

## Abstract

Alterations in eating behavior characterized eating disorders (ED). The genetic factors shared between ED diagnoses have been underexplored. The present study performed a genome-wide association study in individuals with disordered eating behaviors in the Mexican population, blood methylation quantitative trait loci (blood-meQTL), summary data-based Mendelian randomization (SMR) analysis, and in silico function prediction by different algorithms. The analysis included a total of 1803 individuals. We performed a genome-wide association study and blood-meQTL analysis by logistic and linear regression. In addition, we analyzed in silico functional variant prediction, phenome-wide, and multi-tissue expression quantitative trait loci. The genome-wide association study identified 44 single-nucleotide polymorphisms (SNP) associated at a nominal value and seven blood-meQTL at a genome-wide threshold. The SNPs show enrichment in genome-wide associations of the metabolic and immunologic domains. In the in silico analysis, the SNP rs10419198 (*p*-value = 4.85 × 10^−5^) located on an enhancer mark could change the expression of *PRR12* in blood, adipocytes, and brain areas that regulate food intake. Additionally, we found an association of DNA methylation levels of *SETBP1* (*p*-value = 6.76 × 10^−4^) and *SEMG1* (*p*-value = 5.73 × 10^−4^) by SMR analysis. The present study supports the previous associations of genetic variation in the metabolic domain with ED.

## 1. Introduction

Eating disorders (ED) significantly impact individuals’ physical and mental health [1,2,3]. The manifestation of disordered eating behaviors characterizes ED. Disordered eating is the presence of aversive food-related behaviors, including fasting, vomiting, binge eating, and food intake restriction [4,5]. The three primary ED diagnoses are anorexia nervosa, bulimia nervosa, and binge-eating disorder [6]. Individuals diagnosed with ED have different disordered eating patterns, which could change over time [7,8,9]. Even when the diagnosis criteria for ED are well established [6], affected individuals may have a diagnosis crossover [10,11,12]. The diagnosis crossover could result from the close relationship between ED diagnosis criteria and the overlapping of symptoms [8]. Others suggest that this crossover could be an escalation mechanism, mainly in individuals diagnosed with the binge-eating disorder [13].

The etiology of ED is complex, with the interaction between environmental and biological risk factors [14]. Some identified risk factors are genetics, gender, dieting, or weight concerns, occurring early in life or adolescence [15]. In the last decade, different studies have explored the genetic risk factors that underlie many psychiatric disorders, but the analysis of the genetics of ED had been underexplored [16,17]. Furthermore, genome-wide association studies until now had been performed only in individuals diagnosed with AN [18,19,20,21,22]. These studies suggest that AN has a genetic correlation with other psychiatric phenotypes or metabolic traits [18,22]. Nevertheless, the genetic factors shared between ED diagnoses are less explored.

The environment had a role in the etiology of ED. The translation of the environmental risk factors into molecular changes is the focus of epigenetics. DNA methylation is the most studied epigenetic molecular modification, and several studies have reported alterations in DNA methylation in individuals diagnosed with ED [23]. DNA methylation levels depend on the environment and genetic variation, and integrative analysis of genetic variants and DNA methylation levels could identify new biomolecules involved in the etiology of ED. Some authors had developed statistical frameworks to integrate these two sources of genomic information; an example is summary-based Mendelian randomization (SMR) [24]. However, the integration of these two sources of genomic information in individuals diagnosed with ED had not been performed in the Mexican population. The present work aimed to perform a genome-wide association analysis, blood methylation quantitative trait loci (blood-meQTL), summary data-based Mendelian randomization (SMR) analysis, and in silico function prediction in individuals diagnosed with disordered eating behaviors in the Mexican population.

## 2. Materials and Methods

### 2.1. Sample Population

The study included a total of 1803 individuals of Mexican descent, from three different samples: an adolescent clinical subsample from the Mexican Genomic Database for Cross-Disorder Research (*n* = 168, MeDaCrosR) [25,26], a clinical sample recruited from the ED Unit of the Instituto Nacional de Psiquiatría Ramón de la Fuente Muñiz (*n* = 166, INPRFM), and an epidemiological subsample from the Mexican Genomic Database for Addiction Research (*n* = 1469; MxGDAR) [27,28] (Table 1).

Adolescents from the MeDaCrosR were recruited from the Hospital Psiquiátrico Infantil Juan N Navarro (HPIJNN), from the external consultation area. Eating patterns in MeDaCrosR adolescents were evaluated by children’s specialized psychiatrists through the Eating Attitude Test 26 (EAT-26) [29,30] and the Questionnaire on Eating and Weight Patterns Revised (QWEP-R) in Spanish [31]. The adult sample from the INPRFM was diagnosed according to DSM-IV-TR criteria for ED using the Structured Clinical Interview for Mental Disorders v.2.0. (SCID-II) [32] and evaluated with the Eating Disorder Inventory v.2.0. (EDI-2) for psychological patterns [29,30]. The cases of disordered eating were the groups from MeDaCrosR and INPRFM (*n* = 333). The population-based controls (*n* = 1802) were the individuals from the MxGDAR. The Diagnostic Interview for Psychosis and Affective Disorders (DIPAD) screening section was used to evaluate the individuals from MxGDAR [28,33,34,35,36]. The study was performed based in the Helsinki declaration, and was revised by the ethic and investigation committees of the Instituto Nacional de Medicina Genómica (Approval Number CEI/2018/60), Instituto Nacional de Psiquiatría Ramón de la Fuente Muñiz (Approval Number: DGC-279-2008), and the Hospital Psiquiátrico Infantil Juan N Navarro (Approval number II3/01/0913). Every individual fulfilled and signed an informed assent (adolescents) and/or consent (parents/adults).

### 2.2. Genome-Wide Typing

We extracted DNA from blood and buccal epithelial samples with a modified salting-out method, implemented in the commercial kit Gentra Puregene (Qiagen, Readwood City, CA, USA). According to the manufacturer’s protocol, genotyping was performed in the Instituto Nacional de Medicina Genomica with the commercial Infinium PsychArray Beadchip (Illumina, San Diego, CA, USA). Fluorescent intensities were measured with the iScan (Illumina, San Diego, CA, USA), transformed to genotypes with the GenomeStudio (Illumina, San Diego, CA, USA), and converted to Plink format files. Quality control of genotypes was performed in the Plink software [37,38], based on previously published protocols, briefly, we considered the following criteria: variant calling greater than 95%, a minor allele frequency (MAF) greater than 5%, a Hardy–Weinberg equilibrium Chi-square test *p*-value greater than 1 × 10^−5^, and remotion of variants with A/T or G/C alleles (to avoid the flip strand effect). Additionally, we excluded individuals with a genotype call rate of less than 95%. In addition, all individual pairs with an identity-by-state value greater than 1.6 were marked to correct for cryptic relationships, and the individual with the lowest genotype call rate was excluded.

### 2.3. Statistical Analysis

#### 2.3.1. Population Stratification

We evaluated population stratification using previously reported algorithms and the *PC-AiR* package [39]. The reference panel was the Human Genome Diversity Project (HGDP) [40]. We included only independent SNPs by filtering with linkage disequilibrium pruning (LD pruning), using the following parameters: a window size of 50 Kb, a step of 2, and a variance inflation factor of 5 implemented in Plink.

#### 2.3.2. Genome-Wide Associations Analysis

We performed genetic associations through multiple logistic regressions, adjusted for age, sex, and ten components of global ancestry as covariables in Plink. We considered a *p*-value of 5.00 × 10^−5^ as nominally associated, and a *p*-value of 5.00 × 10^−8^ was considered statistically significant on the genome-wide level. After statistical contrasts, we removed all the variants with a MAF lower than 5.0% in cases or controls. The final results included a total of 223,189 SNPs.

#### 2.3.3. Functional Prediction

An in silico functional annotation was also carried out of the associated SNPs using a variant effect predictor (VEP) [41]. A phenome-wide association study was performed in the GWAS atlas of the associated SNPs, and we considered a statistical significance threshold for a *p*-value < 5 × 10^−8^ [42]. Pathway analysis was carried out with the online ComPath tool [43]. Finally, we searched the GTEx portal for in silico multi-tissue expression quantitative trait loci analysis (eQTL) results of the associated variants [44,45].

#### 2.3.4. Methylation Quantitative Trait Loci Analysis

We performed blood methylation quantitative trait loci (blood-meQTL) analysis to estimate the possible impact of the associated SNPs in DNA methylation levels. We analyzed a subset of individuals diagnosed with eating disorders of Mexican ascendence from a previously published database [46]. The DNA methylation levels were determined using Illumina MethylationEPIC BeadChips (Illumina, USA). We calculated *trans*- and *cis*-meQTL with the *-mtscore* function of the KING software [47], and a *p*-value < 5.00 × 10^−8^ was considered genome-wide statistically significant. We searched the associated SNPs with eQTL and blood-meQTL effect in the regulomeDB for enhancer or promoter marks [48].

#### 2.3.5. Summary Data-Based Mendelian Randomization (SMR)

We applied summary data-based Mendelian randomization analysis (SMR) to identify DNA methylation levels associated with disordered eating by pleiotropy [49]. SMR was developed for quantitative expression loci and uses the principles of Mendelian Randomization (MR) to estimate the pleiotropic association between the level of gene expression and a phenotype. Zhu et al. established that if we denote z as a genetic variant (for example, SNP), x as the expression level of a gene, and y as the trait, then the two-step least-squares estimate of the effect of x on y from an MR analysis is:

b_xy_ = b_zy_/b_zx_ (1)

where b_zy_ and b_zx_ are the least-squares estimates of y and x on z, respectively, and b_xy_ is interpreted as the effect size of x on y free of confounding from non-genetic factors. After calculating the sampling variance by the Delta method and replacing b_zy_ and b_zx_ by the estimates, we should have:

(2) $$T_{\mathrm{SMR}}=b_{\mathrm{xy}}^{2}/\mathrm{var}(b_{\mathrm{xy}})=z_{\mathrm{zy}}^{2}\cdot z_{\mathrm{zx}}^{2}/z_{\mathrm{zy}}^{2}+z_{\mathrm{zx}}^{2}$$

where z_zy_ and z_zx_ are the z statistics from the GWAS and eQTL study, respectively. The previous method was developed by Zhu et al., and we used the SMR to integrate the GWAS and blood-meQTL summary statistics.

## 3. Results

In the genotype-phenotype associations, no genome-wide associations were found; nevertheless, 44 single-nucleotide polymorphisms (SNP) were associated at a nominal level (*p*-value < 5 × 10^−5^) (Table 2). The SNPs distributes in 29 cytogenetic bands and 19 coding regions. The protein-coding genes with SNPs associated with disordered eating showed enrichment in the PI3K-Akt signaling pathway (hsa04151, adjusted *p*-value = 0.0499, *FTL3LG* and *TSC2*), vascular muscle contraction (hsa04270, adjusted *p*-value = 0.0494, *KCNMA1* and *PRKCE*), and cGMP–PKG signaling pathway (hsa04022, adjusted *p*-value = 0.0494, *KCNMA1* and *PRKCE*). Of the 44 SNPs, 22 were intronic, and 2 were missense variants. The missense variants were the SRMM4 p.Ser243Asn and FLT3LG p.Phe177Leu.

In the PheWAS, we identified the immunological (rs4626924 and rs10419198) and metabolic (rs3205060, rs8041059 and rs10419198) domain with SNPs associated. The SNPs associated with the immunological domains were both intronic, one located in the coding region of *LOC107985364* and the other in *RCN3*. In the metabolic domain, we identified the rs8041059 located in the intron of *LIPC* and with previous associations with 33 different lipidic traits.

The blood-meQTLs analysis showed seven SNPs associated with DNA methylation levels of five CpG sites (Table 3). The CpG sites map to four genes, including the rs10419198 related to DNA methylation levels in a CpG site annotated to the body of the PRR12. The blood-mQTLs correlated with five CpG sites, annotated to the gene body of *SETBP1* (cg12522870) and *PRR12* (cg06378142), 200 base pairs upstream of the transcription starting site (TSS200) of the non-coding *LOC440704* (cg12412036 and cg09420738) and 1500 upstream of the *SEMG1* (cg15921833). In the case of *LOC440704*, two different CpGs correlated with two SNPs; meanwhile, for the CpGs on *SETBP1* and *SEMG1*, two SNPs connect with the same site.

**Figure 1:**
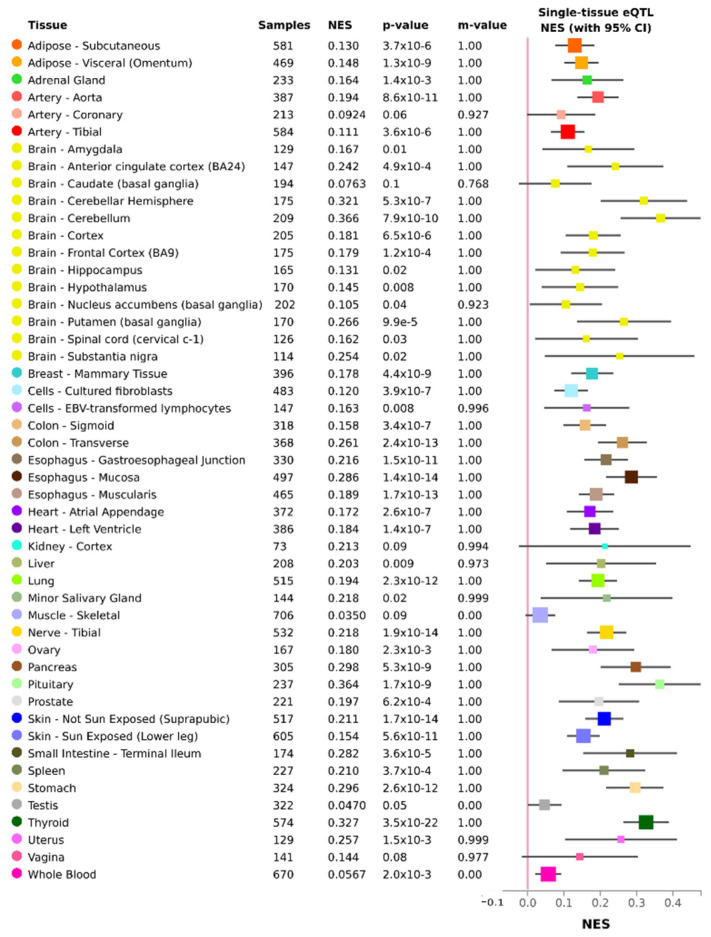
Multi-tissue eQTL (rs10419198) results from the query to GTEx portal. NES is the slope of the linear regression, computed as the effect of the alternative allele relative to the reference allele and m-value is the posterior probability that an effect exists in a tissue.

The multi-tissue eQTL analysis in the GTEx database revealed that the rs10419198 had an eQTL effect on different tissues (frontal cortex, anterior cingulate cortex, cerebellum, putamen, thyroid, visceral adipose tissue, etc.) (Figure 1). In the regulomeDB database, the rs10419198 showed a mark of enhancers in 51 different cell lines.

Of the previous seven SNPs that showed blood-meQTL significant results, we found that two had a pleiotropic effect with DNA methylation levels and disordered eating by SMR analysis (Table 4). The CpG sites were the cg12522870 (*SETBP1*) and cg15921833 (*SEMG1*).

## 4. Discussion

Previous GWAS on ED has shown, mainly in AN, that genetic variants associated with this disorder could significantly impact metabolic pathways [31,32,33,34]. The genetic association found in our study also supports this finding on individuals with disordered eating behavior. In our study, three variants (rs3205060, rs8041059 and rs10419198) associated with disordered eating are previously associated at the genome-wide level with at least one metabolic trait. One associated variant was the rs8041059 which is found in the coding region of the hepatic triglyceride lipase (*LIPC*) and is associated with differences in high-density lipoproteins (HDL), cholesterol, and triglyceride plasmatic levels [35,36]. *LIPC* catalyzes the hydrolysis of triglycerides and phospholipids found in circulating lipoproteins [37,38,39]. Some authors hypothesized that this variant regulates triglyceride concentration, affecting the transcription factors’ binding site in the promoter of *LIPC*. Still, the exact mechanism remains to be explored [40,41,42]. In addition, the association of this SNP could be important for further explorations in the mechanism behind the reported increased HDL levels in individuals diagnosed with AN [43,44].

The other associated variant, rs10419198, is annotated to the intron of *RCN3*, and could function as an enhancer for *PPR12* [36]. Our blood-meQTL and in silico analysis of multi-tissue eQTL showed that this variant modulates the expression of the *PRR12*. *PRR12* is the proline-rich 12 protein, also known as KIAA1205. The exact cellular function of these proteins remains unknown, but it is proposed that it has an essential role in brain development. The role of this gene on the brain is supported by some studies, where individuals with loss-of-function or structural variation on this gene had clinical syndromes characterized by neurodevelopmental and eye abnormalities [45,46,47]. Individuals carriers of high impact variants on *PRR12* had neurodevelopmental disorders, for example, autism, intellectual disability, and attention-deficit/hyperactivity disorder (ADHD). ADHD is one of the most frequent comorbidities found in individuals with ED, and the presence of this clinical entity could increase the symptoms of disordered eating [48,49,50,51]. Further analysis of the function of *PRR12* could help us understand this high comorbidity.

Environmental factors interact with genetics to the development of ED [48]. A known molecular mark that captures these environmental differences is DNA methylation [50]. DNA methylation is influenced by the environment and genetic, having differences based on the genotype of the individuals [51,52]. Then if the DNA methylation level affected the trait, we could see an effect of differences in the phenotype, known as pleiotropy [49,53]. We performed SMR to capture this in our analysis and found an association of disordered eating with CpG sites on *SETBP1* and *SEMG1*. Mutations on *SETBP1* cause the Schinzel Giedion syndrome, individuals affected had a severe intellectual disability. A recent study identified *SETBP1* as a key epigenetic regulator of the gene network responsible for visceral organ and brain morphogenesis [54]. On the other hand, the *SEMG1* could be a key modulator of energy metabolism on ED. *SEMG1* is the semenogelin 1 gene, expressed mainly on the prostate and is suggested to be responsible for asthenozoospermia [55]. In addition, protein pull-down studies suggest that SEMG1 increases energy expenditure by interacting with enzymes responsible for energy regulation, such as lactate dehydrogenase A [56]. Nevertheless, further studies are required to clarify the molecular mechanism of these proteins in ED.

Our study is one of the first to explore genetic associations with ED in the Mexican population at the genome-wide level; nevertheless, we could state some limitations. The main is the small sample size, and also, we do not have a replication cohort. This reduced sample size reduces our statistical power. Another limitation could be the lack of metabolic parameters measured in the sample and that the population-based sample was not screened for disordered eating behavior. On the other hand, integrating different sources of information, the in silico eQTL and blood-meQTL strengthens it.

## 5. Conclusions

The present study suggests an association of the rs10419198 enhancer variant of the *PRR12* supporting previous reports that have found that disordered eating could imply genetic associations with metabolism.

## Figures and Tables

**Table 1:** Overview of the characteristics of the samples.

Characteristic	MeDaCrosR (*n* = 168)	INPRFM (*n* = 166)	MxGDAR (*n* = 1469)
Age, mean (s.d)	13.96 (1.94)	19.32 (4.82)	35.86 (15.77)
Gender			
Male, *n*(%)	42 (0.25)	16 (9.64)	388 (26.41)
Female, *n*(%)	126 (0.75)	150 (90.36)	1081 (73.59)

**Table 2:** Associated SNPs to disordered eating in Mexican population.

SNP	Band	Position	A1/A2	MAF Cases	MAF Controls	OR	L95	U95	*p*-Value	Gene	Effect
rs17030129	1p36.31	1:7059150	A/G	0.3787	0.3145	1.685	1.325	2.141	2.03 × 10^−5^	*CAMTA1*	Intron
rs11120813		1:7062993	A/G	0.4414	0.3720	1.646	1.306	2.075	2.45 × 10^−5^		
rs6690584		1:7078434	G/T	0.4401	0.3645	1.718	1.359	2.170	5.87 × 10^−5^		
rs7521204	1p36.13	1:19138295	T/C	0.5329	0.4193	1.672	1.330	2.100	1.03 × 10^−5^	*Intergenic*	-
rs12024738	1q31.1	1:190694813	A/G	0.5285	0.4238	1.577	1.267	1.964	4.65 × 10^−5^	*LINC01720*	Intron
rs4626924	1q42.3	1:234909298	C/T	0.2260	0.2862	0.5861	0.4536	0.7573	4.38 × 10^−5^	*LOC107985364*	
rs867286	2p21	2:45982030	A/G	0.4505	0.3821	1.655	1.321	2.074	1.18 × 10^−5^	*PRKCE*	Intron
rs11677196	2p12	2:75830221	A/G	0.2949	0.3754	0.5947	0.4688	0.7546	1.83 × 10^−5^	*Intergenic*	-
rs3205060	2q31.1	2:175425346	G/A	0.4249	0.3410	1.657	1.318	2.084	1.57 × 10^−5^	*WIPF1*	3′-UTR
rs7569439	2q35	2:220590633	C/T	0.3091	0.3712	0.57	0.4472	0.7266	5.67 × 10^−6^	*Intergenic*	-
rs35542515		4:161798045	A/C	0.2733	0.2063	1.93	1.472	2.529	1.91 × 10^−6^		
rs2748991	6p12.2	6:52596516	C/T	0.4234	0.3099	1.662	1.303	2.118	4.16 × 10^−5^		
rs3801220	7p14.1	7:42247876	G/A	0.5494	0.4506	1.729	1.379	2.167	2.11 × 10^−6^	*GLI3*	Intron
rs3801232		7:42253313	T/C	0.5284	0.4282	1.778	1.412	2.238	9.70 × 10^−7^		
rs4724100		7:42264679	C/T	0.5254	0.4316	1.726	1.371	2.174	3.50 × 10^−6^		
rs4507768	8q13.3	8:70642018	A/G	0.1272	0.1703	0.5027	0.3635	0.6952	3.23 × 10^−5^	*SLCO5A1*	Intron
rs10114881	9q21.13	9:76676071	T/C	0.5254	0.4298	1.628	1.293	2.049	3.34 × 10^−5^	*Intergenic*	-
rs12241514	10p12.31	10:21602923	A/G	0.1257	0.2088	0.4525	0.3293	0.6219	1.02 × 10^−6^		
rs1865020	10q22.3	10:78688976	C/T	0.4566	0.3764	1.634	1.301	2.052	2.45 × 10^−5^	*KCNMA1*	Intron
rs7918074	10q26.3	10:134277154	A/G	0.2380	0.1547	1.922	1.448	2.551	6.20 × 10^−6^	*LOC105378569*	
rs10870311		10:134290526	A/C	0.3228	0.2279	1.751	1.347	2.275	2.77 × 10^−5^	*Intergenic*	-
rs10772471	12p13.2	12:11600364	A/G	0.3802	0.2754	1.66	1.301	2.117	4.58 × 10^−5^	*LOC440084*	Intron
rs7297606	12q24.3	12:119568596	A/G	0.1886	0.1605	1.918	1.415	2.599	2.66 × 10^−5^	*SRRM4*	Missense (p.Ser243Asn)
rs4075945		12:119569784	T/C	0.1886	0.1609	1.915	1.413	2.599	2.78 × 10^−5^		Intron
rs12809631		12:131045190	A/C	0.1467	0.1954	0.5341	0.3992	0.7146	2.41 × 10^−5^	*RIMBP2*	
rs2144067	14q32.31	14:101952406	T/C	0.2156	0.2330	0.5547	0.4198	0.7330	3.42 × 10^−5^	*Intergenic*	-
rs1007904		14:101955905	A/G	0.2380	0.2589	0.5720	0.4370	0.7488	4.80 × 10^−5^		
rs7163468	15q12	15:26587077	T/C	0.2036	0.1243	1.915	1.399	2.621	4.96 × 10^−5^		
rs3922665		15:26590830	G/A	0.2425	0.1552	1.885	1.413	2.514	1.60 × 10^−5^		
rs8041059	15q21.3	15:58743709	T/C	0.2710	0.1999	1.732	1.329	2.256	4.72 × 10^−5^	*LIPC*	Intron
rs11073665	15q25.3	15:87295120	G/A	0.4027	0.3281	1.626	1.295	2.041	2.78 × 10^−5^	*AGBL1*	
rs17135764	16p13.3	16:2111779	T/C	0.2440	0.3144	0.5455	0.4249	0.7003	1.99 × 10^−6^	*TSC2*	Intron
rs11862729	16p13.12	16:14146098	G/A	0.2395	0.1789	1.809	1.364	2.4000	3.87 × 10^−5^	*Intergenic*	-
rs12454763	18q12.3	18:42434615	A/G	0.4102	0.3341	1.673	1.328	2.108	1.26 × 10^−5^	*SETBP1*	Intron
rs991014		18:42439886	A/G	0.4096	0.3349	1.705	1.350	2.154	7.49 × 10^−6^		
rs1042122	19q13.3	19:49989424	C/T	0.2769	0.3567	0.5677	0.4447	0.7246	5.46 × 10^−6^	*FLT3LG*	Missense (p.Phe177Leu)
rs10419198		19:50038017	T/C	0.3084	0.3833	0.6054	0.4798	0.7638	4.85 × 10^−5^	*RCN3*	Intron
rs6074170	20p12.2	20:10671078	A/G	0.4162	0.3501	1.5940	1.2730	1.9970	4.85 × 10^−5^	*Intergenic*	-
rs4813048		20:11169603	T/C	0.2575	0.1821	1.8440	1.3870	2.4520	2.55 × 10^−5^		
rs6043684	20p12.1	20:16023836	A/C	0.4096	0.3066	1.6330	1.2980	2.0560	2.88 × 10^−5^	*MACROD2*	Intron
rs6104082	20q13.12	20:43897362	C/T	0.3423	0.2827	1.6750	1.3060	2.1470	4.70 × 10^−5^	*LOC105372630*	
rs2824006	21q21.1	21:18099779	C/T	0.4386	0.3584	1.5930	1.2760	1.9900	4.00 × 10^−5^	*Intergenic*	-
rs2824065		21:18187408	C/T	0.3997	0.2968	1.6910	1.3380	2.1370	1.09 × 10^5^		
rs71330155		21:22059184	A/C	0.1587	0.2234	0.5276	0.3922	0.7098	2.38 × 10^−5^		

Note: SNP = single-nucleotide polymorphism, Band = cytogeneitc band, Position = genomic coordinates, A1/A2 = minor allele/major allele frequency, MAF = minor allele frequency, OR = odds ratio, L95 = low 95% confidence interval, U95 = upper 95% confidence interval, Effect = in-silico variant effect prediction.

**Table 3:** Blood methylation quantitative loci (blood-meQTL).

SNP	CpG	Gene	Location	Beta	SE	*p*-Value
rs12024738	cg12412036	*LOC440704*	TSS200	−0.0869	0.0114	2.4216 × 10^−14^
rs12245880	cg09420738			0.1041	0.0155	1.7351 × 10^−11^
rs12454763	cg12522870	*SETBP1*	Body	−0.0775	0.0109	1.6124 × 10^−12^
rs991014				−0.0775	0.0109	1.6124 × 10^−12^
rs10419198	cg06378142	*PRR12*	Body	−0.3768	0.0502	6.2212 × 10^−14^
rs2233903	cg15921833	*SEMG1*	TSS1500	0.2124	0.0153	5.1650 × 10^−44^
rs6104082				0.1895	0.0195	2.3470 × 10^−22^

Note. SNP = single-nucleotide polymorphism, CpG = cytosine to guanine nucleotides, SE = standard error.

**Table 4:** Results of the summary data-based Mendelian randomization.

SNP	CpG	Beta SMR	SE SMR	*p*-Value SMR
rs991014	cg12522870	−1.9889	0.5852	6.7589 × 10^−4^
rs6104082	cg15921833	0.7180	0.2085	5.7263 × 10^−4^

Note. SNP = single-nucleotide polymorphism, CpG = cytosine to guanine nucleotides SE = standard error.

## Data Availability

The data presented in this study are available on request from the corresponding author, omitted due to privacy and ethical issues. The complete summary statistics would be made available as Supplementary Material.

## Supplementary Materials

The following supporting information can be downloaded at: https://www.mdpi.com/article/10.3390/nu14020394/s1, summary statistics of the GWAS of disordered eating.
